# The Relationship Between Spiritual Caregiving and Compassion Levels Among Turkish Nurses: An ICU Case Study

**DOI:** 10.1007/s10943-025-02257-y

**Published:** 2025-02-05

**Authors:** Hülya Fırat Kılıç, Serpil Su, Seda Cevheroğlu

**Affiliations:** 1 Department of Nursing, Faculty of Health Sciences, Eastern Mediterranean University, Via Mersin 10, Famagusta, North Cyprus Turkey; 2 Faculty of Nursing, Necmettin Erbakan University, Konya, Turkey

**Keywords:** Compassion, Spirituality, Spiritual care, Nursing

## Abstract

This descriptive and correlational study aimed to determine the levels of spiritual caregiving and compassion among Turkish Intensive Care Unit (ICU) nurses and evaluate the relationship between these two variables. This study included 135 ICU nurses working in a university hospital in Turkey. Descriptive information forms, the Compassion Scale (CS), and the Spiritual Care-Giving Scale (SCGS) were used for data collection. The participants’ mean CS score was high, with the separation and common humanity subscales yielding the lowest and highest scores, respectively. The mean SCGS score was high, with the lowest and highest scores obtained from the spiritual care attitudes and spirituality perspectives, respectively. There is a positive correlation between CS and SCGS scores. This study concluded that Turkish ICU nurses demonstrated high levels of compassion and spiritual care and that there is a positive relationship between them. Cultural factors can affect compassion and spiritual care; therefore, future studies in different cultures are necessary to provide more valid evidence, possibly through experimental studies.

## Introduction

Compassion is a crucial professional value that nurses worldwide should possess (Durkin et al., 2022). The International Council of Nurses (ICN) acknowledges compassion as a professional value that impacts nurses’ decision-making processes and actions, ultimately contributing to excellence in practice and quality of care (ICN, 2021).

Compassion and spiritual care should be at the center of daily nursing practice for effective, ethical, and holistic nursing care (Dincer & Çiftçi, 2024). Compassion can be defined as “a feeling, attitude or trait that arises in witnessing another’s distress and that motivates a subsequent desire to help” (Sinclair et al., 2017) or “a feeling of pity and sadness as a result of a deep realization of the trauma and pain experienced by individuals” (Bilgiç, 2022). Patients require the most compassion in healthcare facilities and nurses feel this emotion with the greatest intensity (Arkan et al., 2020; Şirin & Yurttaş, 2015). Furthermore, compassion is considered a moral virtue that healthcare providers should exhibit toward patients (Dalgalı & Gürses, 2018). Spirituality, on the other hand, is a “vital principle, a concept that adds spirit, breath, essence, quality and meaning to life and is too broad to be restricted to religion” (Köktürk et al., 2022). With the increasing significance of holistic nursing care, spiritual care has emerged (Çınar & Eti Aslan, 2017; Dincer & Çiftçi, 2024). The concept can be defined as support services offered to inpatients in order to provide spiritual and moral support upon request, to guide them to fulfill their worship within the context of the patient’s illness, ultimately facilitating hope for life (Çınar & Eti Aslan, 2017).

Patients require more compassion and spiritual care in hospitals, which are considered a crisis environment where illness, stress, and fear of death are experienced, and the meaning of life is questioned (Çınar & Eti Aslan, 2017). This need is particularly evident in intensive care units (ICUs), where interventional procedures are frequently performed and mortality and morbidity rates are high, increasing the demand for spiritual support from both patients and their families (Tambağ et al., 2018). Compassionate care and spiritual support in healthcare units have a significant positive impact on patient recovery, comfort, satisfaction, and quality of life (Çınar & Eti Aslan, 2017; Türkben Polat & Özdemir, 2022). However, literature analysis reveals that nurses encounter difficulty in providing adequate spiritual care due to factors such as low levels of compassion, heavy workloads, and inadequate mentorship and knowledge (Chew et al., 2016; Tambağ et al., 2018; Türkben Polat & Özdemir, 2022). Other studies have reported that nurses exhibit positive attitudes toward delivering compassionate spiritual care (Badanta et al., 2022; Eskimez et al., 2022; Taylor et al., 2023).

Compassionate spiritual care provided by ICU nurses can aid the emotional, physiological, and psychological recovery of critically ill patients in the ICU. Therefore, assessing the spiritual caregiving and compassion levels of ICU nurses is crucial. However, no study has explored the relationship between these two concepts. This study aimed to assess the levels of spiritual caregiving and compassion among Turkish ICU nurses and examine the relationship between the two. The research questions were as follows:

- What is the level of compassion in Turkish ICU nurses?
- What is the level of spiritual caregiving provided by Turkish ICU nurses?
- Is there a relationship between compassion and spiritual caregiving in Turkish ICU nurses?

## Methods

### Aim and Design

This descriptive and correlational study aimed to determine the levels of spiritual caregiving and compassion among Turkish ICU nurses and to evaluate the relationship between these two variables.

### Population and Sampling

The study population was comprised of nurses employed in the ICU of a university hospital in Turkey. The adequacy of the sample size was determined using Cohen’s effect size (Cohen, 1988). The correlation between the levels of spiritual care and compassion was ρ H1 = 0.528, with a 95% confidence interval (1–α) and 99% power (1–β), demonstrating that the sample size of 135 was sufficient. This study included volunteer ICU nurses.

### Data Collection Tools

A descriptive information form, Compassion Scale, and Spiritual Care-Giving Scale were used for data collection.

#### Descriptive Information Form

The form was developed by the researchers in line with previous research (Çınar & Eti Aslan, 2017; Türkben Polat & Özdemir, 2022) and comprised 10 questions on age, gender, marital status, education level, professional experience, and years of service in the ICU.

#### Compassion Scale (CS)

The CS was originally developed by Pommier (2011) and was later adapted to Turkish by Akdeniz and Deniz (2016). The scale consists of 24 items measuring six subscales: kindness, indifference, common humanity, separation, mindfulness, and disengagement. Items are scored on a 5-point Likert scale, and the scores obtained from the indifference, separation, and disengagement subscales are reverse-scored. Total scores ranged between one and five, with higher scores indicating higher levels of compassion. The Cronbach’s alpha for the Turkish version of the scale was 0.85 (Akdeniz & Deniz, 2016). Cronbach’s alpha in our study was 0.91.

#### Spiritual Care-Giving Scale (SCGS)

The SCGS was developed by Tiew and Creedy (2012) and adapted to Turkish by İpek Çoban et al. (2017). This 35-item scale consists of five subscales: spirituality, general properties of spiritual care, spiritual care attitudes, defining spiritual care, and spiritual care practices. Higher scores indicated higher levels of spirituality and perceptions of spiritual care (İpek Çoban et al., 2017; Tiew & Creedy, 2012). The Cronbach’s alpha values of our study and the Turkish version of the scale were 0.97 and 0.96, respectively.

### Data Collection Process

The participants were informed of the study’s purpose, and verbal and written informed consent was obtained from all participants. After informing the participants that their participation was voluntary and that data confidentiality was assured, they were asked to complete the data collection tools. Data were collected during face-to-face interviews, which lasted approximately 15 min.

### Statistical Analysis

SPSS (Statistical Package for the Social Sciences) version 26.0 (IBM Corp., Armonk, NY, USA) was used for statistical analysis. Frequency (%) was used to present qualitative variables, whereas the median and interquartile range (P25%–P75%) were used to present continuous variables. The normality of the scores obtained for continuous variables was tested using the Kolmogorov–Smirnov test. The Mann–Whitney *U* test was used to compare two groups, whereas the Kruskal–Wallis *H* test was used to compare more than two groups. The Mann–Whitney *U* test was performed to compare two groups, whereas the Kruskal–Wallis *H* test was used to compare more than two groups. The Spearman’s correlation test was performed to examine the correlation between two quantitative variables. A multivariate linear regression model was used to determine the independent variables associated with the dependent variables. Statistical significance was set at *p* < 0.05.

### Ethical Considerations

Ethical approval was obtained from the Scientific Research and Publication Ethics Committee of the Eastern Mediterranean University (No. ETK00-2022–0297). Institutional permission was obtained from the Meram Medical Faculty Hospital at Necmettin Erbakan University. Written informed consent was obtained using a voluntary informed consent form prepared in accordance with the principles of the Declaration of Helsinki.

## Results

### Descriptive Characteristics

The median age of the 135 participants was 30 years (IQR, 26–33), 68% were female, 60% were married, 84% had a bachelor’s degree or above, 52% had professional experience exceeding five years and 34% had more than five years of experience in ICU nursing. In addition, 84% of the ICU nurses worked 40–49 h weekly, 83% worked night shifts, and 74% preferred ICUs as their place of work (Table 1).

**Table 1:** Descriptive characteristics

Variables (*N* = 135)	*n* (%)
*Age, median*(*P*_25_–*P*_75_)	30(26–33)
< 30 years	67(49.6)
≥ 30 years	68(50.4)
*Gender*	
Female	92(68.1)
Male	43(31.9)
*Marital status*	
Married	81(60)
Single	54(40)
*Education*	
Vocational school of health (VSH)	9(6.7)
Associate degree	12(8.9)
Undergraduate degree	103(76.3)
Graduate degree	11(8.1)
*Professional experience*	
≤ 5 years	65(48.1)
6–10 years	31(23)
≥ 11 years	39(28.9)
*ICU stage*	
1^st^Stage	6(4.4)
2nd Stage	12(8.9)
3rd Stage	117(86.7)
*ICU experience*	
≤ 5 years	89(65.9)
> 5 years	46(34.1)
*Weekly working hours*	
40–49 h	114(84.4)
≥ 50 h	21(15.6)
*Night shifts*	
Yes	112(83)
No	23(17)
*Prefers to work in the ICU*	
Yes	100(74.1)
No	19(14.1)
Doesn’t matter	16(11.8)

### Compassion Levels

This study assessed the compassion levels of Turkish ICU nurses using the CS. The mean and median CS scores were 3.95 ± 0.56 and 4 (IQR, 3.5–4.4), respectively. Participants obtained the lowest and highest scores from the separation (2.06 ± 0.69) and common humanity subscales (4.05 ± 0.63), respectively (Table 2). An analysis of compassion levels based on descriptive characteristics showed that female participants scored significantly higher on the indifference (*Z* = -3.137; *p* = 0.002) and disengagement (*Z* = -2.104; *p* = 0.035) subscales, but obtained significantly lower scores on the total CS (*Z* = -2.348; *p* = 0.019). In addition, nurses working in third-stage ICUs exhibited higher levels of mindfulness (*Z* = -2.600; *p* = 0.009) (Table 3). Finally, nurses who worked less than 50 h a week and preferred to work in ICUs scored higher on the common humanity subscale of the CS (*Z* = -2.175, *p* = 0.030 and *Z* = -2.368, *p* = 0.018, respectively) (Table 4).

**Table 2:** Scores obtained from the scales and subscales

Scale	Mean (SD)	Median (IQR)
**CS Total**	3.95(0.56)	4(3.5–4.4)
Kindness	3.99(0.74)	4(3.5–4.8)
Indifference*	2.19(0.79)	2.3(1.5–2.8)
Common humanity	4.05(0.63)	4.3(3.8–4.5)
Separation*	2.06(0.69)	2(1.5–2.5)
Mindfulness	4.00(0.66)	4(3.5–4.5)
Disengagement*	2.10(0.72)	2(1.5–2.5)
**SCGS Total**	4.10(0.64)	4(3.7–4.7)
General properties of spiritual care	4.11(0.71)	4.1(3.6–4.8)
Spirituality perspectives	4.14(0.72)	4.3(3.8–4.8)
Defining spiritual care	4.00(0.71)	4(3.6–4.6)
Spiritual care practices	4.13(0.69)	4(3.8–4.8)
Spiritual care attitudes	3.97(0.73)	4(3.3–4.7)

IQR: Inter Quantile Range(P_25%_–P_75%_), SD: Standard deviation,

^*^Reverse-scored

**Table 3:** Correlation between the CS and SCGS scores

		No											
No	Variables	1	2	3	4	5	6	7	8	9	10	11	12
1	**CS Total**	N/A											
2	Kindness	.859^*^											
3	Indifference	− .842^*^	− .589^*^										
4	Common humanity	.656^*^	.617^*^	− .354^*^									
5	Separation	− .824^*^	− .637^*^	.716^*^	− .478^*^								
6	Mindfulness	.772^*^	.687^*^	− .560^*^	.473^*^	− .490^*^							
7	CS-Disengagement	− .838^*^	− .633^*^	.784^*^	− .382^*^	.685^*^	− .540^*^						
8	**SCGS Total**	.528^*^	.518^*^	− .304^*^	.499^*^	− .485^*^	.404^*^	− .443^*^					
9	General properties	.457^*^	.468^*^	− .219^***^	.462^*^	− .420^*^	.357^*^	− .382^*^	.932^*^				
10	Spirituality perspectives	.578^*^	.557^*^	− .388^*^	.486^*^	− .517^*^	.430^*^	− .473^*^	.859^*^	.700^*^			
11	Defining spiritual care	.390^*^	.403^*^	− .185^***^	.391^*^	− .359^*^	.322^*^	− .300^*^	.850^*^	.724^*^	.752^*^		
12	Spiritual care practices	.530^*^	.478^*^	− .337^*^	.523^*^	− .513^*^	.418^*^	− .409^*^	.917^*^	.858^*^	.744^*^	.766^*^	
13	Spiritual care attitudes	.369^*^	.390^*^	− .195^***^	.349^*^	− .338^*^	.291^**^	− .346^*^	.820^*^	.756^*^	.672^*^	.708^*^	.797^*^

^*^p < 0.001, **p < 0.01, ***p < 0.05, Spearman’s correlation, **N/A:** Not available

**Table 4:** CS scores according to descriptive characteristics

	Kindness	Indifference	Common humanity	Separation	Mindfulness	Disengagement	CS Total
Variables	Median (IQR)	Median (IQR)	Median (IQR)	Median (IQR)	Median (IQR)	Median (IQR)	Median (IQR)
*Age*							
< 30 years	4.0[3.3–4.5]	2.0[1.5–2.8]	4.0[3.5–4.5]	2.0[1.8–2.5]	4.0[3.5–4.5]	2.0[1.5–2.5]	4.0[3.4–4.4]
≥ 30 years	4.3[3.5–4.8]	2.3[1.6–2.8]	4.3[3.8–4.5]	2.0[1.5–2.5]	4.0[3.5–4.8]	2.0[1.5–2.5]	4.0[3.5–4.5]
Test^a^/*P*-value	− *1.646/0.100*	− *0.575/0.565*	− *0.237/0.812*	− *0.862/0.389*	− *0.727/0.467*	− *0.193/0.847*	− *0.619/0.536*
*Gender*							
Female	4.0[3.5–4.8]	2.0[1.5–2.5]	4.3[3.8–4.5]	2.0[1.5–2.5]	4.0[3.5–4.5]	1.8[1.5–2.5]	4.1[3.5–4.5]
Male	4.0[3.3–4.5]	2.5[2.0–3.0]	4.0[3.3–4.5]	2.3[1.8–2.5]	3.8[3.3–4.5]	2.3[2.0–2.8]	3.8[3.5–4.0]
Test^a^/*P*-value	− *1.467/0.142*	− 3.137/0.002*	− *1.449/0.147*	− *1.319/0.187*	− *1.056/0.291*	− 2.104/0.035*	− 2.348/0.019*
*Marital status*							
Married	4.0[3.5–4.5]	2.3[1.5–2.9]	4.3[3.8–4.5]	2.0[1.5–2.5]	4.0[3.6–4.5]	2.0[1.5–2.8]	4.0[3.5–4.4]
Single	4.0[3.3–4.8]	2.0[1.5–2.6]	4.1[3.7–4.8]	2.0[1.8–2.5]	3.9[3.5–4.5]	2.0[1.5–2.5]	4.1[3.5–4.4]
Test^a^/*P*-value	− *0.446/0.656*	− *0.970/0.332*	− *0.713/0.476*	− *0.407/0.684*	− *0.701/0.483*	− *0.300/0.764*	− *0.288/0.774*
*Education*							
VSH/Associate	4.3[3.1–4.6]	2.0[1.5–2.8]	4.3[3.5–4.8]	2.0[1.8–2.5]	3.8[3.4–4.6]	2.0[1.8–2.6]	4.0[3.5–4.4]
Undergraduate/Graduate	4.0[3.5–4.8]	2.3[1.7–2.8]	4.1[3.8–4.5]	2.0[1.5–2.5]	4.0[3.5–4.5]	2.0[1.5–2.5]	4.0[3.5–4.4]
Test^a^/*P*-value	− *0.394/0.693*	− *0.985/0.325*	− *0.499/0.618*	− *0.394/0.693*	− *0.217/0.828*	− *0.208/0.836*	− *0.237/0.813*
*Professional experience*							
≤ 5 years	3.8[3.3–4.5]	2.0[1.5–2.9]	4.0[3.5–4.5]	2.0[1.8–2.5]	4.0[3.5–4.5]	2.0[1.5–2.5]	3.9[3.4–4.4]
6–10 years	4.3[4.0–4.5]	2.3[2.0–3.0]	4.3[4.0–4.8]	2.0[1.5–2.5]	4.0[3.5–4.5]	2.3[1.5–2.8]	4.0[3.5–4.4]
≥ 11 years	4.0[3.5–4.8]	2.0[1.5–2.5]	4.3[3.8–4.5]	2.0[1.5–2.5]	4.0[3.5–5.0]	2.0[1.3–2.5]	4.1[3.7–4.6]
Test^b^/*P*-value	*4.732/0.094*	*4.010/0.135*	*3.817/0.148*	*2.069/0.355*	*2.264/0.322*	*2.934/0.231*	*2.576/0.276*
*ICU stage*							
1/2.Stage	4.4[3.9–5.0]	1.8[1.5–2.6]	4.0[3.7–5.0]	2.0[1.3–2.5]	4.6[3.8–5.0]	1.8[1.2–2.1]	4.2[3.8–4.6]
3.Stage	4.0[3.3–4.5]	2.3[1.5–2.8]	4.3[3.8–4.5]	2.0[1.5–2.5]	4.0[3.5–4.5]	2.3[1.5–2.5]	4.0[3.5–4.4]
Test^a^/*P*-value	− *1.649/0.099*	− *1.359/0.174*	− *0.509/0.611*	− *0.867/0.386*	− 2.600/0.009*	− *1.784/0.074*	− *1.839/0.066*
*ICU experience*							
≤ 5 years	4.0[3.3–4.6]	2.0[1.5–2.8]	4.0[3.6–4.5]	2.0[1.5–2.5]	4.0[3.5–4.5]	2.0[1.5–2.5]	4.0[3.5–4.4]
> 5 years	4.3[3.5–4.8]	2.3[1.5–2.8]	4.3[3.8–4.5]	1.9[1.5–2.5]	4.0[3.3–4.8]	2.3[1.5–2.8]	3.9[3.6–4.5]
Test^a^/*P*-value	− *1.375/0.169*	− *0.212/0.832*	− *0.299/0.765*	− *0.734/0.463*	− *0.552/0.581*	− *0.074/0.940*	− *0.529/0.595*
*Weekly working hours*							
40–49 h	4.0[3.5–4.8]	2.0[1.5–2.8]	4.3[3.8–4.5]	2.0[1.5–2.5]	4.0[3.5–4.5]	2.0[1.52.5]	4.0[3.5–4.4]
≥ 50 h	4.0[3.0–4.8]	2.3[1.9–2.9]	4.0[3.1–4.3]	2.3[1.6–2.5]	3.8[3.5–4.4]	2.3[1.5–3.0]	3.8[3.3–4.4]
Test^a^/*P*-value	− *0.636/0.525*	− *1.095/0.274*	− 2.175/0.030*	− *0.700/0.484*	− *0.544/0.586*	− *1.203/0.229*	− *1.215/0.224*
*Night shifts*							
Yes	4.0[3.3–4.5]	2.3[1.8–2.8]	4.0[3.8–4.5]	2.0[1.5–2.5]	4.0[3.5–4.5]	2.3[1.5–2.5]	4.0[3.5–4.4]
No	4.3[3.8–4.8]	1.5[1.0–3.0]	4.3[3.5–4.8]	2.0[1.3–2.8]	4.3[3.8–5.0]	1.8[1.3–2.5]	4.3[3.4–4.8]
Test^a^/*P*-value	− *1.583/0.114*	− *1.647/0.100*	− *0.723/0.470*	− *0.533/0.594*	− *1.237/0.216*	− *1.948/0.051*	− *1.467/0.142*
*Prefers to work in the ICU*							
Yes	4.0[3.5–4.8]	2.0[1.5–2.8]	4.3[3.8–4.5]	2.0[1.5–2.5]	4.0[3.5–4.5]	2.0[1.5–2.5]	4.0[3.6–4.4]
No/Doesn’t matter	4.0[3.3–4.5]	2.3[1.8–3.3]	4.0[3.5–4.3]	2.3[1.8–2.8]	4.0[3.5–4.5]	2.3[1.8–2.5]	3.8[3.4–4.4]
Test^a^/*P*-value	− *1.001/0.317*	− *1.940/0.052*	− 2.368/0.018*	− *1.483/0.138*	− *0.751/0.453*	− *0.947/0.344*	− *1.660/0.097*

^*^p < 0.05, a: Mann–Whitney U test, b: Kruskal Wallis-H test, IQR: Inter Quantile Range (P_25%_–P_75%_)

### Spiritual Caregiving

The SCGS was used to evaluate the levels of spiritual caregiving provided by participating Turkish ICU nurses. The mean and median SCGS scores were 4.10 ± 0.64 and 4 (IQR, 3.7–4.7), respectively. Participants obtained the lowest and highest scores from the subscales of spiritual care attitudes (3.97 ± 0.73) and spirituality perspectives (4.14 ± 0.72) (Table 2). There were no statistically significant differences between the SCGS scores and descriptive characteristics (*p* > 0.05). (Table 5).

**Table 5:** SCGS scores according to descriptive characteristics

	General properties	Spirituality perspectives	Defining spiritual care	Spiritual care practices	Spiritual care attitudes	SCGS Total
Variables	Median (IQR)	Median (IQR)	Median (IQR)	Median (IQR)	Median (IQR)	Median (IQR)
*Age*						
< 30 years	4.0[3.7–4.9]	4.1[3.8–4.6]	4.0[3.6–4.6]	4.0[3.8–4.8]	4.0[3.3–4.7]	4.0[3.7–4.7]
≥ 30 years	4.2[3.5–4.7]	4.3[3.8–4.6]	4.0[3.5–4.6]	4.1[3.8–4.8]	4.0[3.4–4.7]	4.0[3.7–4.7]
Test^a^/*P*-value	− *0.272/0.786*	− *0.424/0.671*	− *0.011/0.991*	− *0.175/0.861*	− *0.847/0.397*	− *0.126/0.900*
*Gender*						
Female	4.0[3.6–4.9]	4.1[3.8–4.8]	4.0[3.8–4.8]	4.0[3.8–4.8]	4.0[3.3–4.7]	4.0[3.7–4.8]
Male	4.3[3.7–4.7]	4.3[3.5–4.6]	4.0[3.4–4.4]	4.0[3.8–4.8]	4.0[3.7–4.7]	4.2[3.7–4.8]
Test^a^/*P*-value	− *0.624/0.533*	− *0.716/0.474*	− *0.741/0.459*	− *0.167/0.868*	*1.273/0.203*	− *0.196/0.845*
*Marital status*						
Married	4.2[3.7–4.9]	4.3[3.8–4.8]	4.0[3.6–4.6]	4.0[4.0–4.8]	4.0[3.3–4.7]	4.1[3.8–4.7]
Single	4.0[3.5–4.7]	4.1[3.7–4.6]	4.0[3.4–4.6]	4.0[3.7–4.5]	4.0[3.7–4.4]	4.0[3.7–4.6]
Test^a^/*P*-value	− *1.272/0.203*	− *0.857/0.391*	− *0.808/0.419*	− *1.117/0.264*	− *0.278/0.781*	− *1.166/0.243*
*Education*						
VSH/Associate	4.0[3.3–4.7]	4.0[3.4–4.6]	3.8[2.9–4.4]	4.0[3.5–4.6]	4.0[3.3–4.7]	3.9[3.6–4.5]
Undergraduate/Graduate	4.1[3.6–4.9]	4.3[3.8–4.8]	4.0[3.6–4.6]	4.1[3.8–4.8]	4.0[3.7–4.7]	4.1[3.7–4.7]
Test^a^/*P*-value	− *1.024/0.306*	− *1.092/0.275*	− *1.694/0.090*	− *0.827/0.408*	− *0.397/0.692*	− *1.157/0.247*
*Professional experience*						
≤ 5 years	4.0[3.5–4.8]	4.1[3.8–4.6]	4.0[3.6–4.6]	4.0[3.7–4.8]	3.7[3.3–4.3]	3.9[3.7–4.6]
6–10 years	4.2[3.8–4.8]	4.4[3.9–4.6]	4.0[3.8–4.6]	4.2[4.0–4.8]	4.0[3.7–4.7]	4.1[3.8–4.7]
≥ 11 years	4.2[3.7–5.0]	4.3[3.6–5.0]	4.0[3.4–4.4]	4.2[3.8–4.8]	4.0[3.3–5.0]	4.1[3.7–4.8]
Test^b^/*P*-value	*0.905/0.636*	*0.392/0.822*	*0.889/0.641*	*1.567/0.457*	*2.477/0.290*	*0.752/0.687*
*ICU stage*						
1/2.Stage	4.2[3.6–4.9]	4.4[3.8–4.6]	4.0[3.8–4.7]	4.3[3.8–5.0]	4.0[3.7–4.7]	4.3[3.7–4.8]
3.Stage	4.0[3.6–4.8]	4.1[3.8–4.8]	4.0[3.6–4.5]	4.0[3.8–4.8]	4.0[3.3–4.7]	4.0[3.7–4.7]
Test^a^/*P*-value	− *0.298/0.843*	− *0.654/0.513*	− *0.849/0.396*	− *0.758/0.449*	− *1.026/0.305*	− *0.508/0.611*
*ICU experience*						
≤ 5 years	4.0[3.5–4.8]	4.3[3.8–4.8]	4.0[3.6–4.6]	4.0[3.8–4.8]	4.0[3.5–4.7]	4.0[3.7–4.7]
> 5 years	4.2[3.8–4.8]	4.2[3.5–4.7]	4.0[3.4–4.4]	4.2[4.0–4.8]	4.0[3.3–4.7]	4.0[3.7–4.7]
Test^a^/*P*-value	− *0.562/0.574*	− *0.543/0.587*	− *0.604/0.546*	− *0.124/0.901*	− *0.729/0.466*	− *0.095/0.924*
*Weekly working hours*						
40–49 h	4.0[3.6–4.8]	4.3[3.8–4.7]	4.0[3.6–4.5]	4.0[3.8–4.8]	4.0[3.3–4.7]	4.0[3.7–4.6]
≥ 50 h	4.1[3.6–4.8]	4.1[3.8–5.0]	4.0[3.7–5.0]	4.3[3.8–4.8]	4.0[3.3–4.7]	4.0[3.8–4.8]
Test^a^/*P*-value	− *0.280/0.779*	− *0.531/0.596*	− *1.489/0.136*	− *0.640/0.522*	− *0.209/0.834*	− *0.586/0.558*
*Night shifts*						
Yes	4.1[3.6–4.8]	4.1[3.8–4.6]	4.0[3.6–4.6]	4.0[3.8–4.8]	4.0[3.7–4.7]	4.0[3.7–4.6]
No	4.0[3.8–5.0]	4.4[3.9–5.0]	4.0[3.4–4.8]	4.0[4.0–5.0]	4.0[3.3–5.0]	4.0[3.8–5.0]
Test^a^/*P*-value	− *0.394/0.694*	− *1.217/0.224*	− *0.318/0.751*	− *0.540/0.589*	− *0.344/0.731*	− *0.597/0.550*
*Prefers to work in the ICU*						
Yes	4.1[3.6–4.9]	4.3[3.8–4.8]	4.0[3.6–4.6]	4.2[3.8–4.8]	4.0[3.3–4.7]	4.0[3.7–4.8]
No/Doesn’t matter	4.0[3.7–4.5]	4.1[3.5–4.6]	4.0[3.6–4.4]	4.0[3.7–4.5]	4.0[3.7–4.3]	4.0[3.7–4.5]
Test^a^/*P*-value	− *0.804/0.421*	− *0.789/0.430*	− *0.482/0.630*	− *1.261/0.207*	− *0.267/0.789*	− *0.716/0.474*

*p* > 0.05, a: Mann–Whitney U test, b: Kruskal Wallis-H test, IQR: Inter Quantile Range(P_25%_–P_75%_)

### Correlation Between Continuous Variables

There was a positive and statistically significant correlation between the CS and SCGS scores, indicating that an increase in compassion was associated with an increase in The SCGS was used to evaluate the levels of spiritual caregiving provided by participating Turkish ICU nurses. ICU nurses’spiritual caregiving (*r* = 0.528; *p* < 0.001) (Table 3).

### Independent Factors Affecting Spiritual Caregiving

A multiple linear regression model was used to identify the independent variables that affected the levels of spiritual caregiving among Turkish ICU nurses (*F *_*(6–128)*_ = 11.159, *p* < 0.001). The analysis of the relationship between the independent and dependent variables showed that indifference (β1 = -0.286, β2 = -0.352, *p* = 0.007) and disengagement (β1 = -0.327, β2 = -0.352, *p* = 0.004) subscales of the CS decreased spiritual caregiving while common humanity (β1 = 0.280, β2 = 0.274, *p* = 0.004) increased spiritual caregiving (Table 6).

**Table 6:** Independent factors affecting spiritual caregiving

Variables	β1	SE	95% CI		β2	t	*p*	VIF	Tolerance	F	DW Statistics
			Lower	Upper						*p*	AdjR^2^
(Constant)	2.698	0.541	1.628	3.768		4.989	< 0.001				
CS-Kindness	0.044	0.105	− 0.164	0.252	0.050	0.418	0.677	2.830	0.354		
CS-Indifference	− 0.286	0.103	− 0.490	− 0.081	− 0.352	− 2.760	0.007*	3.170	0.315	11.159	2.080
CS-Common humanity	0.280	0.095	0.092	0.468	0.274	2.951	0.004*	1.680	0.595	0.001*	0.313
CS-Separation	− 0.327	0.111	− 0.548	− 0.107	− 0.352	− 2.940	0.004*	2.800	0.357		
CS-Mindfulness	0.125	0.102	− 0.076	0.326	0.129	1.230	0.221	2.140	0.467		
CS-Disengagement	− 0.174	0.115	− 0.401	0.053	− 0.195	− 1.519	0.131	3.200	0.313		

^*^p < 0.05, β1 = Estimates of unstandardized regression weights, β2 = Estimates of standardized regression weights, SE = Standard error, CI: Confidence interval, Multivariate Linear Regression Analysis Results

## Discussion

This study, which was conducted to determine the relationship between compassion and spiritual caregiving levels of Turkish ICU nurses, found that the participants had high levels of compassion and spiritual caregiving, and that the level of spiritual caregiving increased as the compassion level of nurses increased. We did not find any other studies analyzing the relationship between levels of spiritual caregiving and compassion among ICU nurses. The findings of this study have been discussed in the relevant literature.

The compassion levels of Turkish ICU nurses in this study were relatively high. Similarly, other studies have noted high levels of compassion among nurses (Arkan et al., 2020; Eskimez et al., 2022; Tanrıkulu & Ceylan, 2021; Türkben Polat & Özdemir, 2022). Patients require compassion in health care facilities and anticipate that nurses will provide compassionate care (Arkan et al., 2020). Providing patient-centered and humane care is challenging for nurses without a sense of compassion (Tanrıkulu & Ceylan, 2021). Thus, our research indicated that the respondents delivered comprehensive and humanistic care.

In our study, female participants displayed higher levels of compassion than their male counterparts did. The literature review indicates that some studies did not identify a significant relationship between gender and compassion (Arkan et al., 2020; Erdem & Uyaroğlu, 2021; Eskimez et al., 2022; Tanrıkulu and Ceylan (2021); ; ; ; ; ; ; ; ; ; ; ; ;;;;;;, while others reported a higher level of compassion among female nurses (Çingöl et al., 2018; Gündüzoğlu et al., 2019; Kaçan, 2023). Our finding is expected as it is attributed to the emotional nature and maternal instincts exhibited by female nurses (Buldur & Göçen, 2021). In addition, it is thought that these gender-related characteristics are effective in the higher indifference and disengagement scores in men in this study.

In the study, the level of mindfulness of nurses working in third-stage ICUs was found to be lower. Mindfulness is the individual’s balanced approach to negative emotions (Neff, 2003). In this regard, it can be said that nurses working in third-stage ICUs have difficulty controlling their emotions. The reason for this may be the complex emotions experienced by nurses working here while caring for patients with complicated health problems. Because the rate of multiple organ failure and mortality is higher in patients treated in the third-stage ICUs (ICUSC, 2008). According to the results of this study, it is of great importance to support nurses working in third-stage ICUs in terms of being aware of and controlling emotions.

In this study, it was found that the level of common humanity was high in nurses who worked less than 50 h a week and preferred to work in in ICUs. We think that these results were obtained due to the high level of empathy in these nurses. Because being common humanity is the individual’s awareness that suffering is a common experience of all humanity. With the feeling of compassion, he perceives the suffering individuals as part of a common life rather than independent of him, and this is related to empathy (Neff, 2003). Arkan et al. (2020) reported that intensive care nurses who used empathy during patient care had higher levels of compassion and common humanity. This result is consistent with our study finding and emphasizes the creation of working conditions that support empathy for care that includes compassion.

In this study, it was determined that the Turkish ICU nurses had a high level of spiritual caregiving. An analysis of the literature reveals that spiritual caregiving has not been accurately measured, with most studies employing instruments to measure attitudes, beliefs, and perceptions of spiritual care (Merati-fashi et al., 2021). Despite positive attitudes toward spiritual care, nurses rarely provide it (Taylor et al., 2023). This study enhanced the scholarly discourse by measuring the level of spiritual caregiving among ICU nurses, a topic previously overlooked in the literature. This gap in the literature improves the value of this study because it determined the level of spiritual caregiving in ICU nurses, which has not been previously measured. Previous studies have utilized the SCGS to measure spiritual caregiving in ICU nurses (Ramadhan et al., 2020), acute care nurses (Chew et al., 2016) and nursing students (Köktürk et al., 2022) and reached similar conclusions. There are other studies that utilized other scales and reported moderate (Akgün Şahin & Kardaş Özdemir, 2016; Türkben Polat and Özdemir (2022); ; ; ; ; or low levels of spiritual caregiving (Mamier et al., 2019).

This study found a positive correlation between levels of compassion and spiritual caregiving. Although no studies had previously been conducted on ICU nurses, other studies have reported comparable results. Türkben Polat and Özdemir (2022) found that compassion accounted for 31% of spiritual care therapeutics. Kaçan (2023) reported a positive correlation between the levels of perceived compassion and spiritual caregiving. Similarly, Dincer noted a positive relationship between compassion competencies and perceptions of spirituality and spiritual care in nursing students (Dincer & Çiftçi, 2024). Akin et al. (2021) discovered a proportional rise in levels of compassion with an enhancement in spiritual caregiving among midwives. Considering that compassion and spirituality are at the center of nursing care, the findings of this study may be positively evaluated in terms of increasing the quality of care of patients.

The indifference and disengagement subscales of the CS negatively correlated with spiritual caregiving, whereas common humanity increased it. Spiritual care is founded on compassion and unconditional love and is naturally influenced by nurses’ characteristics (Kaçan, 2023). According to previous research, factors such as nurses’ level of education, knowledge, skills, and the amount of time devoted to patients can impact the level of spiritual care (Taylor et al., 2023). These characteristics are particularly crucial in ICUs, where nurses administer personalized care. However, previous studies have not thoroughly investigated the impact of compassion on spiritual care. Our findings can serve as a guiding framework for developing nursing care plans in ICUs.

### Limitations

This study had two limitations. Firstly, almost all of the nurses participating in the study work in the third-stage ICUs, and this may have affected the study results. The second limitation is that the study results cannot be generalized because it was conducted in a single center and was based on self-report.

## Conclusion

This study examined the correlation between levels of compassion and spiritual caregiving among Turkish ICU nurses. This study found that the levels of both were relatively high and positively correlated. In addition, it was found that the level of compassion of female nurses was higher, the level of mindfulness was lower in nurses working in third-stage ICUs, and the level of common humanity was higher in nurses who worked less than 50 h a week and preferred to work in intensive care. According to these results, since compassion and spiritual care are affected by cultural characteristics, similar studies should be conducted in different cultures; conducting studies in which the number of nurses working in primary, secondary and third-stage ICUs is similar; it is recommended to conduct comparative studies in hospitals with different patient profiles and to provide stronger evidence through experimental studies.
